# ASQ-PHI: An adversarial synthetic data benchmark for clinical de-identification and search utility

**DOI:** 10.1016/j.dib.2026.112586

**Published:** 2026-02-11

**Authors:** James Weatherhead, George Golovko, Peter McCaffrey

**Affiliations:** a Graduate School of Biomedical Sciences, University of Texas Medical Branch (UTMB), 301 University Boulevard, Galveston, TX 77555, USA; b Department of Pharmacy, University of Texas Medical Branch (UTMB), 301 University Boulevard, Galveston, TX 77555, USA; c Department of Pathology, University of Texas Medical Branch (UTMB), 301 University Boulevard, Galveston, TX 77555, USA

**Keywords:** Protected health information (PHI), Information retrieval, Clinical text anonymization, HIPAA Safe Harbor, Synthetic clinical text, Privacy-preserving information retrieval, Health informatics

## Abstract

Hospitals and vendors now run HIPAA-compliant Business Associate Agreement (BAA) large language models (LLMs) for clinical work. These systems do not use input data for further training, so clinicians can enter Protected Health Information (PHI) into them. LLMs are trained on a fixed corpus with a historical cutoff, therefore their answers often need to be supplemented with more recent clinical evidence from external sources such as live web search or other tools that are often not covered by a BAA. This creates a “safe handoff” point where a clinician’s PHI-containing query must be transformed into a HIPAA Safe Harbor compliant version before leaving the protected environment. However, publicly shareable datasets for this setting are scarce; this article describes PHI-rich clinician-style questions paired with HIPAA Safe Harbor annotations at the point where an external tool is called. Existing de-identification benchmarks are typically built from long electronic health record narratives such as discharge summaries and clinic notes, rather than from short, compressed search-style queries such as those that might be used in chat-based clinical LLM interfaces. ASQ-PHI (Adversarial Synthetic Queries for Protected Health Information de-identification) is a fully synthetic benchmark dataset designed for this safe handoff setting; no real patient data, electronic health records, or protected health information were accessed, used, or referenced during dataset creation. It contains 1051 single-turn clinical search queries that are designed to resemble prompts that clinicians might enter into HIPAA-compliant LLMs. Each record uses machine-parsable delimiters to separate the free text query from PHI annotations, which are provided as one JSON object per element specifying the HIPAA Safe Harbor identifier category and exact string value. The corpus includes 832 PHI-positive queries (79.2%) and 219 hard negatives (20.8%) engineered to mimic PHI-like syntax while containing only non-identifying clinical information such as ages under 90 years, diagnoses, medications, and symptoms. Across the dataset, there are 2973 PHI elements labeled from 13 textual HIPAA Safe Harbor identifier types that can be represented as short alphanumeric strings in single-line clinical questions, supporting the measurement of both PHI removal and over-redaction on PHI-free queries. All queries were generated with an adversarial few-shot prompting pipeline using Azure OpenAI GPT-4o. The associated Mendeley Data repository provides the complete dataset file, a Jupyter notebook that implements the generation pipeline, summary statistics, baseline metrics for a commercial PHI detection service, and six figures that describe the dataset. ASQ-PHI is released under an MIT license.

Specifications Table

**Table:** 

Subject	Health Sciences, Medical Sciences & Pharmacology
Specific subject area	Synthetic HIPAA Safe Harbor PHI dataset of clinician-style search queries
Type of data	Plain-text corpus, CSV/TXT tables, PNG figures, and processed baseline metric files
Data collection	1051 fully synthetic clinical search queries generated and tagged via adversarial few-shot prompting of Azure OpenAI GPT-4o, with tagged JSON annotations for HIPAA Safe Harbor categories. Hard negative queries are explicitly engineered to mimic PHI-containing structure while containing no PHI. Baseline de-identification metrics are computed using Amazon Comprehend Medical (DetectPHI) [1] at confidence thresholds 0.0, 0.3, 0.5, and 0.8. No real patient data, electronic health records, or identifiable clinical information were accessed, used, or referenced during dataset creation; all patient names, dates, locations, medical record numbers, and other identifiers are machine-generated.
Data source location	University of Texas Medical Branch at Galveston, Galveston, Texas, United States
Data accessibility	Instructions for accessing these data: Openly available in Mendeley Data under an MIT license. Repository name: Mendeley Data Data identification number: 10.17632/csz5dzp7nx.1 Direct URL to data: https://data.mendeley.com/datasets/csz5dzp7nx/1 Code repository: https://github.com/JamesWeatherhead/asq-phi
Related research article	None

## 1 Value of the data

• This dataset [2] provides 1051 fully synthetic single-turn clinician-style queries, each paired with JSON annotations for 13 HIPAA Safe Harbor identifier types, at the ‘safe handoff’ boundary where HIPAA BAA LLMs call external tools, allowing PHI-redaction system developers and LLM platform teams to evaluate leakage and over-redaction on realistic queries.
• Existing de-identification corpora are built mainly from long narrative EHR documents such as discharge summaries and clinic notes [3], rather than the short, compressed questions that clinicians enter into HIPAA-aligned LLM chat interfaces [4]. ASQ-PHI provides a query-style benchmark for this setting and is formatted to resemble the questions that would leave the BAA-protected environment.
• The dataset is intentionally adversarial, combining PHI-dense queries with structurally similar hard-negative queries that contain no identifiers, so that de-identification systems can be evaluated for both PHI leakage and over-redaction at the BAA-to-external-tool handoff; the prompt design follows established few-shot prompting practices and uses GPT-4o as the generator model [5,6].
• Each query is paired with machine-parsable JSON tags for Safe Harbor identifiers [7] that can be expressed as short alphanumeric strings or phrases in single-line clinical queries (for example names, dates, sub-state locations, and medical record numbers). The dataset also includes 219 carefully engineered hard negatives that mimic PHI-like syntax while containing only non-identifying clinical content, which supports simultaneous measurement of missed PHI and over-redaction on PHI-free inputs.
• The data are stored as a single plain-text file with fixed delimiters and one JSON object per PHI element. This layout is straightforward to parse in most programming languages and is compatible with a wide range of NLP workflows, including sequence tagging, masking or redaction, rule-based pattern induction, and evaluation of single- or multi-agent LLM systems that must decide when and how to de-identify queries before tool calls.
• ASQ-PHI is fully synthetic, contains no real patient information, and is released under an MIT license with an accompanying Jupyter notebook that documents the generation pipeline, PHI-tag schema, and validation checks. The notebook enables users to generate new datasets with the same structure and annotation schema, including domain-specific variants (for example oncology, pediatrics, or telemedicine); however, due to the stochastic nature of LLM generation, re-running the pipeline will produce different query content while maintaining format. The dataset has been audited by independent clinicians for clinical plausibility and PHI label correctness, which supports reuse as a benchmark by NLP researchers, LLM platform teams, PHI-redaction vendors, and compliance or security groups.

## 2 Background

Hospitals deploying HIPAA-compliant LLM systems [4] and exploring external tools, for example live web search, often rely on de-identification corpora built from long EHR narratives, such as discharge summaries and clinic notes [3,[8], [9], [10]], rather than short, query-style inputs such as those that might be used in BAA-protected clinical LLM chat interfaces. At this “safe handoff” boundary, PHI-containing queries must be de-identified under the HIPAA Safe Harbor standard [7] before any content leaves the BAA environment, but publicly shareable PHI-rich query-style text for this setting is not widely available. Obtaining real queries from production LLM logs typically involves institutional approvals, IRB oversight, and governed systems, which affects their availability as open benchmarks. ASQ-PHI was compiled in this context using a GPT-4o generation pipeline to create fully synthetic, information-seeking questions annotated with HIPAA Safe Harbor identifier types. LLM-generated synthetic datasets have been used for NLP benchmarking [11], and synthetic clinical corpora have been described as substitutes for restricted real clinical data [12,13]. The accompanying code records the prompts, generation workflow, and validation procedures used to construct and audit the ASQ-PHI dataset.

## 3 Data Description

The ASQ-PHI Mendeley repository [2] is organized with four folders at the top level (code, data, figures, validation_results) and one dependency file (requirements.txt). Table 1 provides an overview of the Mendeley data repository, followed by file-level detail.

**Table 1:** Overview of folders and files in the ASQ-PHI repository [2].

Folder / file	Type	Content
data/synthetic_clinical_ queries.txt	Plain text	Core ASQ-PHI dataset. 1051 synthetic clinical search queries with PHI annotations in a fixed delimiter format.
data/dataset_statistics.txt	Plain text	Summary statistics for the dataset, including counts of queries, PHI elements, PHI types, and PHI density per query.
code/data_generation_ pipeline.ipynb	Jupyter notebook	End-to-end generation pipeline used to create ASQ-PHI from an Azure OpenAI GPT-4o deployment.
code/.env.example	Template text file	Example environment variables for configuring the Azure OpenAI client and output path.
figures/fig1_data_ structure_example.png	PNG image	Schematic example of one query block with its PHI tags.
figures/fig2_phi_type_ distribution.png	PNG image	Bar chart of PHI counts by HIPAA Safe Harbor identifier type.
figures/fig3_dataset_ composition.png	PNG image	Pie chart showing the proportion of PHI-positive queries versus hard negatives.
figures/fig4_phi_density_ distribution.png	PNG image	Histogram of the number of PHI elements per query.
figures/fig5_leakage_recall_ baseline.png	PNG image	Figure of recall and number of leaked PHI entities across confidence thresholds for one baseline de-identification system.
figures/fig6_overredaction_ baseline.png	PNG image	Figure of specificity and over-redaction rate for hard negatives across the same thresholds on the baseline de-identification system.
validation_results/positive_ metrics_by_threshold.csv	CSV	Threshold-wise de-identification metrics for PHI-positive queries, derived from the baseline de-identification system.
validation_results/negative_ metrics_by_threshold.csv	CSV	Threshold-wise de-identification metrics for hard negative queries, derived from the same baseline.
requirements.txt	Text	Python package dependencies required to run the notebook.

The core dataset is the file data/synthetic_clinical_queries.txt. It stores all 1051 synthetic clinical search queries as alternating blocks of query text and PHI annotations. Each record begins with a line that contains the delimiter string ===QUERY===, followed by a single line that contains the entire query. The next delimiter line, ===PHI_TAGS===, marks the start of the annotation block. That block consists of zero or more JSON objects, one per line, of the form {"identifier_type": "TYPE", "value": "…"}. The identifier_type field takes values drawn from a fixed set aligned with HIPAA Safe Harbor, including identifiers such as NAME, GEOGRAPHIC_LOCATION, DATE, MEDICAL_RECORD_NUMBER, HEALTH_PLAN_BENEFICIARY_NUMBER, PHONE_NUMBER, SOCIAL_SECURITY_NUMBER, EMAIL_ADDRESS, UNIQUE_IDENTIFIER, ACCOUNT_NUMBER, FAX_NUMBER, CERTIFICATE_LICENSE_NUMBER, and IP_ADDRESS. The value field repeats the exact string span from the query. These 13 categories were selected to represent Safe Harbor identifiers that can be expressed as short alphanumeric strings or phrases in single-line search queries. The remaining Safe Harbor elements that depend on non-text modalities or long serial patterns (for example full-face photographs, biometric patterns, or device and vehicle identifiers) were not modeled. This format is intentionally simple: developers can reconstruct token-level labels, span boundaries, or masking schemes by parsing a single text file rather than learning a new format.

The queries in synthetic_clinical_queries.txt fall into two structural categories that matter for evaluation. Most entries are PHI-positive queries where the annotation block contains at least one JSON line. The remainder are hard negatives that mimic the phrasing of PHI-containing queries but have an empty PHI tag block. The companion file data/dataset_statistics.txt summarizes this composition in human-readable form. It reports the total number of queries, the number and percentage of PHI-positive queries and hard negatives, the total count of PHI elements, and the mean and median number of PHI elements per query. It also provides the frequency of each identifier type and the distribution of queries with 0, 1, 2, 3, 4 or 5 tagged PHI elements. These same quantities are visualized in the figures folder so that readers can see how dense and how diverse the PHI tags are. By pairing each short query with either PHI-bearing content or a carefully constructed hard negative that mimics PHI-like syntax, ASQ-PHI provides a consistent positive-negative structure.

The code folder contains the generation and validation logic as a single Jupyter notebook, data_generation_pipeline.ipynb, plus a configuration template .env.example. The notebook reads Azure OpenAI credentials from environment variables, instantiates an Azure OpenAI GPT-4o client and defines a long system prompt that enforces the required query style and PHI-tagging rules. It exposes a function generate_phi_queries which sends batched chat completion requests and appends each batch to an output file in the exact ASQ-PHI format. A second function, validate_dataset, parses any such file, counts valid records, recomputes the number and proportion of PHI-positive and hard negative queries, totals the PHI annotations and calculates mean PHI per query. The validation routine also checks that the hard negative proportion, PHI density and malformed-record rate fall within predefined bounds and prints a short quality report. Users can use this notebook as a template to follow the original generation procedure, adapt the prompts, and create similarly structured datasets or domain-specific variants with minimal additional code. The .env.example file lists the expected environment variables, including API key, endpoint, deployment name and optional output path.

The remaining two figures, fig5_leakage_recall_baseline.png and fig6_overredaction_baseline.png, are derived from the baseline validation outputs in the validation_results folder. The file positive_metrics_by_threshold.csv reports, for each confidence threshold of Amazon Comprehend Medical’s DetectPHI [1], the entity-level recall on the PHI-positive subset and the corresponding number of ground-truth PHI entities that remain unmasked. Recall is calculated as TP/(TP + FN), where TP is the number of correctly redacted PHI entities and FN is the number that remain unmasked (reported as leaked_phi_entities in the CSV). The file negative_metrics_by_threshold.csv reports, for the same thresholds, the query-level specificity on PHI-free hard negative queries and the complementary over-redaction rate. Specificity is calculated as TN/(TN + FP), where TN is the number of hard negative queries with no predicted PHI and FP is the number with at least one false-positive redaction; the over-redaction rate is 1 - specificity. Fig. 5 plots recall and leaked-entity counts across thresholds using separate y-axes, and Fig. 6 plots specificity and over-redaction rate across the same thresholds. These plots are descriptive visualizations of the CSV files in the repository and provide an example of how outputs from one widely used PHI detector [1] can be summarized on this dataset.

Finally, the root-level file requirements.txt lists the Python packages needed to run data_generation_pipeline.ipynb. It specifies the OpenAI Python client (openai==1.75.0) and the tqdm progress-bar utility (tqdm==4.67.1). Installing these requirements in a Python 3.12 environment, creating a .env file based on .env.example, and executing the notebook allows readers to follow the documented generation procedure and apply the validation workflow. The included dataset file (`data/synthetic_clinical_queries.txt`) serves as the canonical benchmark; re-running the generation code will produce new queries with different content due to LLM sampling stochasticity.

## 4 Experimental Design, materials and Methods

The ASQ-PHI dataset was generated using Python 3.12 and a single Jupyter notebook stored as code/data_generation_pipeline.ipynb. All Python dependencies are listed in requirements.txt. The notebook assumes access to an Azure OpenAI Service GPT-4o deployment and reads configuration from environment variables defined in a .env file in the code/ directory, templated in code/.env.example. The required variables are AZURE_OPENAI_API_KEY_4o, AZURE_OPENAI_ENDPOINT_4o, AZURE_OPENAI_DEPLOYMENT_4o, and an optional OUTPUT_PATH that defaults to synthetic_clinical_queries.txt in the project root.

Within the notebook, the Azure OpenAI client is initialized using the AzureOpenAI class from the openai package, and we used the API version 2023–07–01-preview. Global parameters define the generation behavior: TEMPERATURE is set to 0.9 and BATCH_SIZE is set to 5. A higher temperature was chosen to encourage diverse, adversarial phrasing of queries while keeping outputs coherent and on format, after pilot runs at lower temperatures produced more repetitive structures and fewer PHI patterns. The constant variable AZURE_OPENAI_DEPLOYMENT stores the GPT-4o deployment name, and OUTPUT_PATH controls the location of the output text file. Progress during the generation is tracked using the tqdm library.

**Fig. 1:**
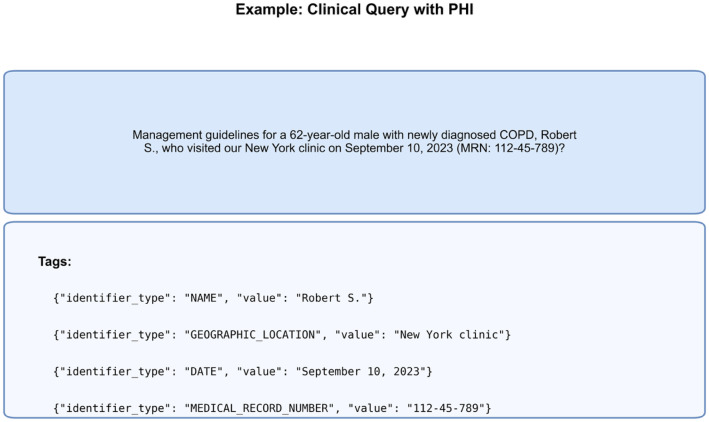
Example ASQ-PHI clinical query and corresponding PHI tag format.

The core design of the dataset is encoded in a single system prompt string, PHI_QUERY_SYSTEM_PROMPT. This prompt instructs the model to act as a clinical data simulation assistant that generates synthetic clinical search queries suitable for evaluating automated HIPAA Safe Harbor de-identification. It constrains the style of queries to information-seeking questions that clinicians might pose to an LLM deployed under a HIPAA BAA, rather than electronic health record-retrieval commands. The prompt defines a strict output format in which each output consists of a query block beginning with the delimiter ===QUERY=== and a PHI annotation block beginning with ===PHI_TAGS===. The query text must be a single line without internal line breaks. The PHI block either contains at least one JSON object, each with keys identifier_type and value, for every identifier present in the query, as shown in Fig. 1, or be left empty for hard negative examples, containing no PHI. The prompt explicitly lists the HIPAA Safe Harbor identifier types that may be tagged and instructs the model not to tag non-identifying clinical information such as diagnoses, medications, or eponymous disease names. The system prompt enumerates the full set of 18 HIPAA Safe Harbor categories for completeness, but given the short, text-only nature of the synthetic queries in this dataset, generation and validation yielded annotations solely from the 13 textual identifier types (e.g., excluding non-textual elements like biometrics or photographs, as detailed in the Data Description). This configuration was intended to focus on query-relevant PHI and to discourage obviously implausible content. It also specifies that the dataset should contain both PHI-dense queries with at least two identifiers and “hard negative” queries that resemble PHI-containing prompts but contain no identifiers and therefore must have an empty PHI block. This setup follows the general few-shot prompting paradigm for in-context learning in large language models [5] and leverages the documented capabilities of the GPT-4 model family [6].

Synthetic queries are generated by the function generate_phi_queries. This function takes the desired number of records, n, and an optional output path. It creates the output directory if needed, then divides the requested total into batches of size five, where the batch size is the number of complete QUERY/PHI_TAGS records requested from GPT-4o in a single API call. In this implementation BATCH_SIZE is set to 5, so each call asks the model to return five queries and their PHI tags at once. This value was chosen after pilot runs as a practical compromise: it is small enough that the combined output of several records fits comfortably within the max_tokens = 2500 limit without truncation, large enough to keep the total number of API calls manageable, and it limits the effect of any malformed or off-format response to at most five records rather than hundreds in a single call. Users who modify BATCH_SIZE should adjust max_tokens in tandem to ensure that the model has enough capacity to return all requested records, delimiters, and JSON tags in a single response. For each batch, the function sends a chat completion request to the GPT-4o deployment. The request uses the system message set to PHI_QUERY_SYSTEM_PROMPT and a user message asking for BATCH_SIZE new, unique clinical queries with structured PHI tags, as specified in the prompt. The temperature parameter is fixed at 0.9, as described earlier, and max_tokens is set to 2500 based on pilot runs as the smallest setting that avoided response truncation for five query-plus-PHI-tag records (one single-line query and several JSON tag lines per record). The function extracts the generated text from the response object. If the response contains the string ===QUERY===, it strips trailing whitespace and appends the batch output, followed by a blank line, to the file at OUTPUT_PATH. If a batch response is malformed or an exception occurs, the function logs the error to generation_errors.log and continues with the next batch. After all batches have run, the function prints the number of successful batches and, if the output file exists, calls the validation routine on that file.

**Fig. 2:**
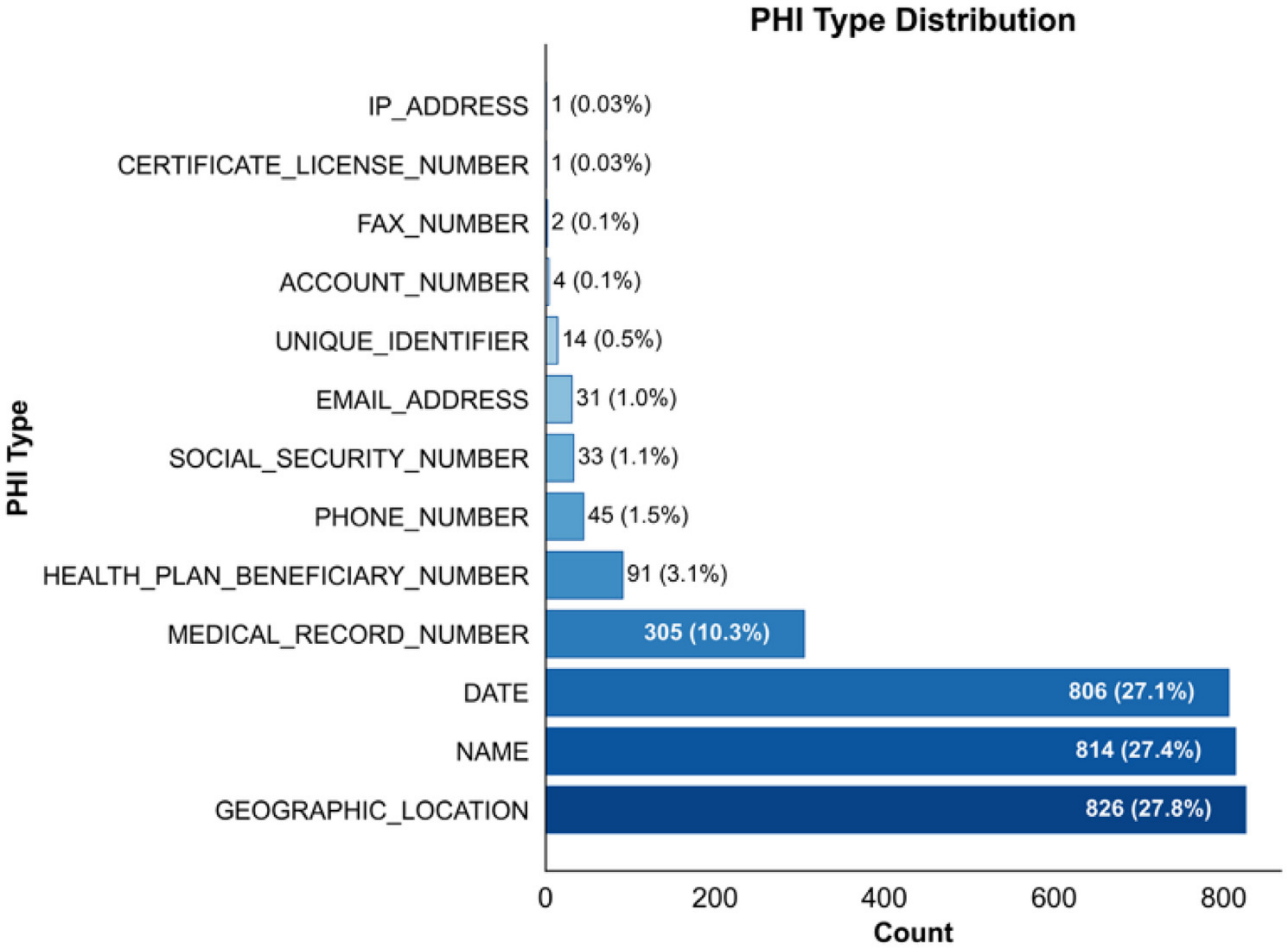
Distribution of HIPAA PHI identifier types in the ASQ-PHI corpus.

**Fig. 3:**
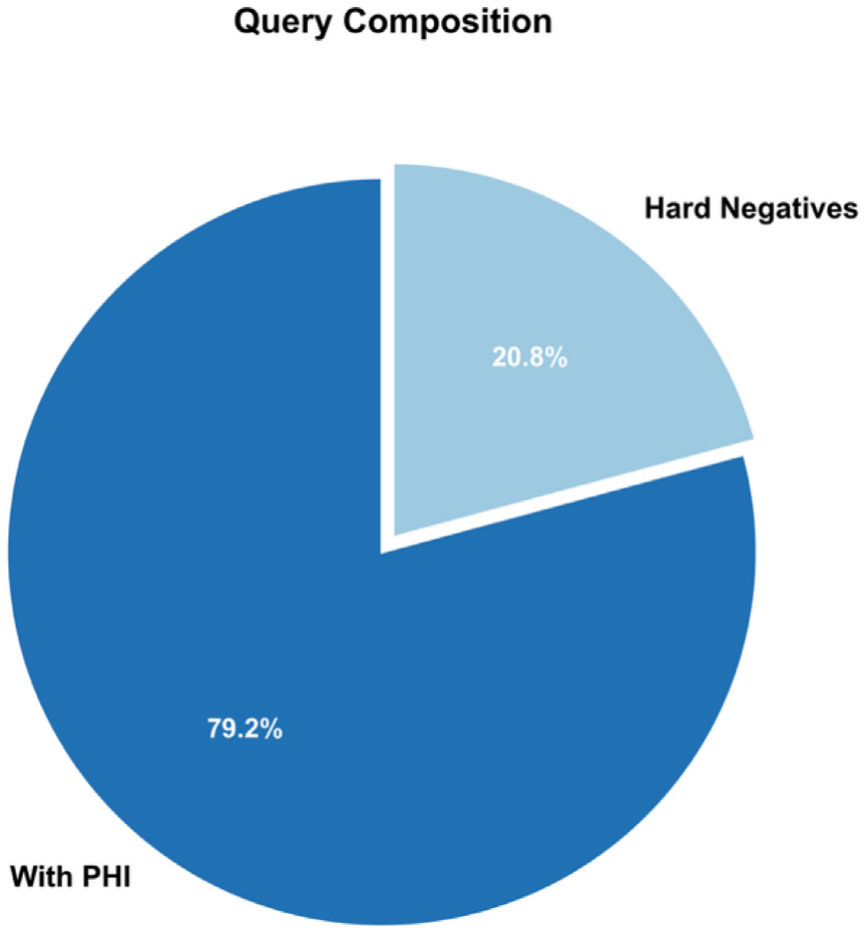
Proportion of PHI-positive queries versus hard negative queries.

**Fig. 4:**
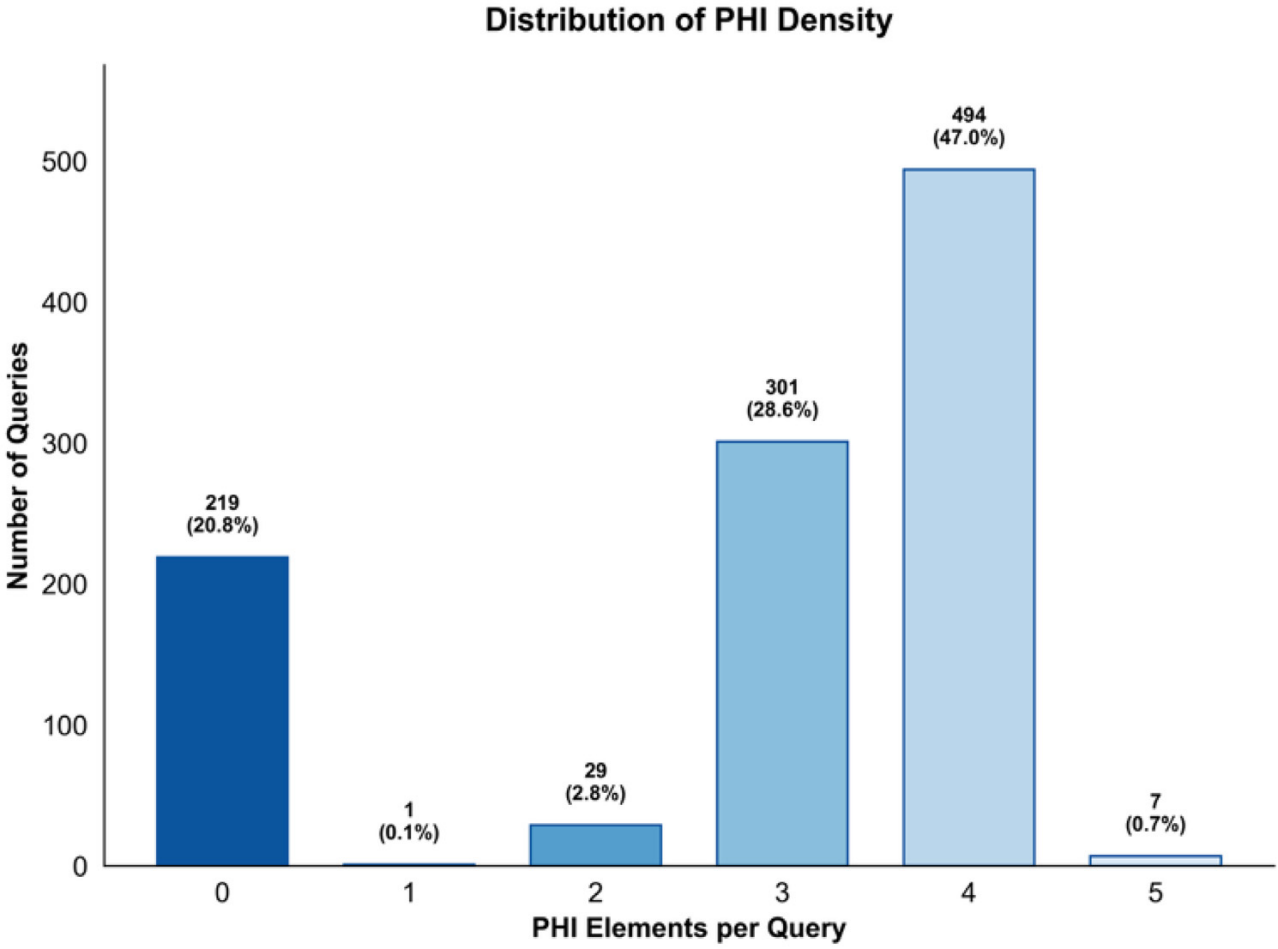
Distribution of the number of PHI elements per query in ASQ-PHI.

The file synthetic_clinical_queries.txt is the primary experimental output. Each record can be parsed by splitting on the delimiter ===QUERY=== and then separating the single-line query text from the subsequent ===PHI_TAGS=== section. Within the PHI section, each JSON object is a single-line tag with identifier_type and value fields, drawn from the 13 textual HIPAA Safe Harbor categories modeled in this dataset (for example NAME, GEOGRAPHIC_LOCATION, DATE, MEDICAL_RECORD_NUMBER, HEALTH_PLAN_BENEFICIARY_NUMBER, PHONE_NUMBER, SOCIAL_SECURITY_NUMBER, EMAIL_ADDRESS, UNIQUE_IDENTIFIER, ACCOUNT_NUMBER, FAX_NUMBER, CERTIFICATE_LICENSE_NUMBER, and IP_ADDRESS). Fig. 1 gives a visual example of one QUERY/PHI_TAGS block. From this text file we computed the higher-level dataset statistics, including the total count of 1051 queries, the split into 832 PHI-positive queries and 219 hard negatives, the total of 2973 PHI elements, the per-query PHI density, and the distribution of PHI types across the 13 categories; these summary values are recorded in data/dataset_statistics.txt and used as the source for Figs. 2, 3, and 4.

**Fig. 5:**
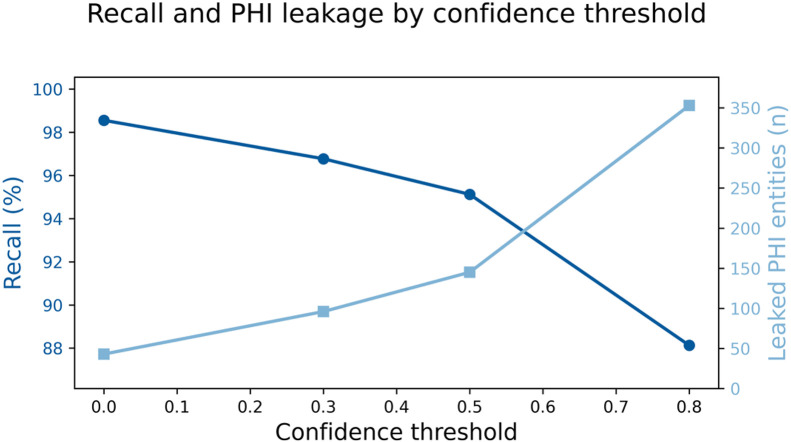
Entity-level recall and number of leaked PHI entities for Amazon Comprehend Medical DetectPHI across confidence thresholds on PHI-positive queries [1].

**Fig. 6:**
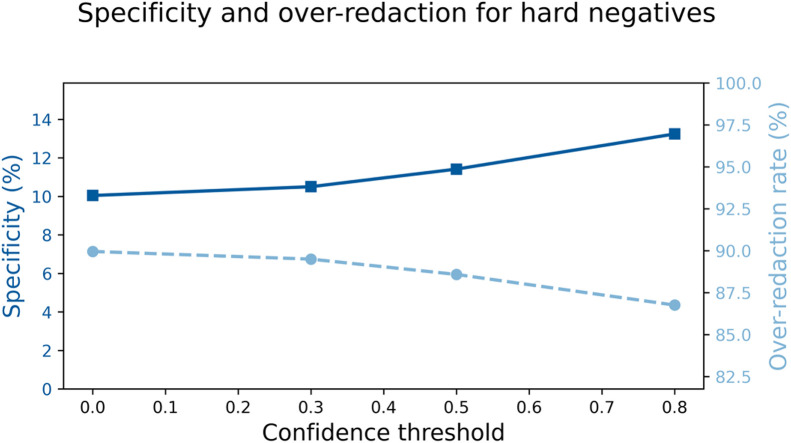
Query-level specificity and over-redaction rate for Amazon Comprehend Medical DetectPHI across confidence thresholds on PHI-free hard negative queries [1].

Dataset quality control is implemented in the function validate_dataset. This function reads the entire contents of a candidate output file, splits it on the ===QUERY=== delimiter, and for each resulting block checks for the presence of ===PHI_TAGS===. Blocks without this second delimiter are counted as malformed. For blocks that contain both delimiters, the function separates the query text from the PHI section. Records with non-empty query text are counted as valid queries. Within the PHI section, non-empty lines that begin with a left brace character are counted as PHI elements. Queries that contain at least one such line are classified as PHI-positive; queries with no PHI lines are classified as hard negatives. The function then computes the number of valid queries, the number and percentage of PHI-positive queries and hard negatives, the total number of PHI elements, the mean number of PHI elements per valid query, and the number of malformed entries. It applies three automated quality checks: whether the hard negative proportion falls between 15 and 35 % of the total dataset, whether the mean PHI per query falls between 1.5 and 5, and whether the malformed rate is below 5 %. These ranges are explicit engineering design choices rather than literature-derived thresholds: a 15 to 35 % hard negative rate was chosen to ensure that de-identification systems encounter a meaningful number of realistic PHI-free queries without letting non-PHI cases dominate the corpus, a mean of 1.5 to 5 PHI elements per query was selected to keep individual queries neither trivially sparse nor unrealistically overloaded with identifiers, and a malformed record rate below 5 % sets a practical ceiling on noise introduced by synthetic generation. The function prints a validation report with these counts, flags each quality check as passed or failed, returns a dictionary of the computed metrics, and its output for the final synthetic_clinical_queries.txt file is what populates data/dataset_statistics.txt and underlies Figs. 2–4.

The repository also includes baseline validation outputs for Amazon Comprehend Medical’s DetectPHI [1] in the validation_results directory. These CSV files were produced by applying DetectPHI to all queries in synthetic_clinical_queries.txt at several confidence thresholds and comparing its predictions to the ground truth PHI annotations in the dataset. The file positive_metrics_by_threshold.csv contains the summary of precision, recall, and related metrics for the subset of PHI-positive queries, and the file negative_metrics_by_threshold.csv contains summary statistics describing predictions on the hard negative subset, where any predicted PHI indicates over-redaction. The figures fig5_leakage_recall_baseline.png and fig6_overredaction_baseline.png were generated from these CSV files. The scripts used to call Amazon Comprehend Medical’s DetectPHI [1] are thin wrappers around the public API and contain deployment-specific configuration and credentials, so they are not included in the deposited record; instead, we provide the resulting CSV outputs and figures, which are sufficient to reproduce the baseline curves and threshold-wise metrics from the underlying dataset. Researchers who would like an example evaluation script may contact the corresponding author.

Finally, the notebook describes a procedure for creating domain-specific variants of ASQ-PHI using the same experimental design. Users are instructed to edit the three example queries in section 7 of the PHI_QUERY_SYSTEM_PROMPT to reflect a particular clinical domain, such as oncology, cardiology, pediatrics, emergency medicine, or telemedicine, while preserving the overall instructions about PHI tagging, query structure, and hard negatives. After modifying these few-shot examples, users rerun generate_phi_queries with a desired sample size and reapply validate_dataset to confirm that the new synthetic dataset meets the same quality thresholds for hard negative ratio, PHI density, and malformed records. This procedure yields a new benchmark with the same file structure, annotation schema, and validation checks, though generated query content will vary due to LLM sampling stochasticity.

Three independent reviewers (board-certified clinicians or equivalent domain experts) each evaluated a random sample of 100 records (total audited *n* = 300) to validate clinical plausibility of the underlying QUERY text and PHI label correctness. This subsample size is justified as a precision-based quality estimate for a binomial outcome: with *n* = 100 per reviewer, an observed plausibility rate near 0.95 yields a 95 % confidence interval with an absolute margin of error of approximately ±4–5 %, and pooling across reviewers (*n* = 300) tightens precision to roughly ±2–3 %, providing a stable estimate of overall dataset validity. In addition, an audit of 300 records has >99 % power at α=0.05 to reject an unacceptably low plausibility floor (for example ≤0.80) when true plausibility is high (≥0.90), and *a* ≥ 95 % chance of detecting any systematic error mode with ≥3 % prevalence. Reviewers scored each record on two dimensions: (i) clinical plausibility of the QUERY text and scenario, defined as whether the question reads as a sensible, answerable clinician-style query independent of how realistic the synthetic names, dates, or institutions are, and (ii) correctness of the PHI_TAGS JSON labels for all Safe Harbor identifier types present in the QUERY. Across the 300 audited records, 96 % of queries were rated clinically plausible (95 % confidence interval 93–98 %), 98 % of PHI labels were judged correct, and 4 of 300 records (1.3 %) were re-generated after consensus review to correct minor inconsistencies between the QUERY and its PHI_TAGS, such as ambiguous temporal phrases that did not map cleanly to a single DATE tag.

## Limitations

ASQ-PHI is built to test a specific failure point, the safe handoff from BAA LLMs to external tools, rather than to mirror the full diversity of real clinician behavior. All queries are fully synthetic, English only, and generated by a single GPT-4o prompting pipeline, so the phrasing, PHI structures, and clinical scenarios inevitably reflect the model’s biases and the prompt design. The dataset does not estimate real-world query frequencies, specialty mix, or true PHI prevalence. Performance on ASQ-PHI reflects effectiveness of PHI redaction on short query-style inputs but does not, on its own, establish suitability for production deployment; teams should complement it with domain-specific validation to ensure clinical utility and compliance.

## Ethics Statement

The authors have read and follow the ethical requirements for publication in Data in Brief and confirm that the current work does not involve human subjects, animal experiments, or any data collected from social media platforms. All queries and Protected Health Information annotations in the ASQ-PHI dataset are fully synthetic and generated by a Large Language Model (Azure OpenAI GPT-4o). No real patient data, electronic health records, or other protected health information were accessed, used, or referenced during dataset creation. Institutional Review Board approval was not required as no human subjects were involved in this research.

## CRediT authorship contribution statement

**James Weatherhead:** Conceptualization, Methodology, Software, Data curation, Writing – original draft, Visualization. **George Golovko:** Supervision, Validation, Formal analysis, Writing – review & editing. **Peter McCaffrey:** Supervision, Writing – review & editing.

## Data Availability

Mendeley DataASQ-PHI: An Adversarial Synthetic Benchmark for Clinical De-Identification and Search Utility (Original data)
